# Design of the Pore Structure of Sponge-Structured Cement Pastes with Both Absorption and Storage Functions

**DOI:** 10.3390/ma18245537

**Published:** 2025-12-10

**Authors:** Tong Li, Guojun Du, Hefang Zhang, Dongli Wang, Xiangwang Tao, Jinqiu Zhang

**Affiliations:** 1 Key Laboratory of Mechanical Reliability for Heavy Equipment and Large Structures of Hebei Province, Yanshan University, Qinhuangdao 066004, China; 2 Bohai Rim Energy Research Institute, Northeast Petroleum University, Qinhuangdao 066004, China; 3 College of Civil Engineering and Architecture, Northeast Petroleum University, Daqing 163318, China15663122926@163.com (J.Z.)

**Keywords:** cement-based materials, pore structure, capillary water absorption performance, water storage performance, cement paste, mineral admixture

## Abstract

This study uses fly ash and slag as the main raw materials to replace 80% of the cement, and prepares a sponge-structured cement paste with storage and absorption functions. This paste is then used to bind the coarse aggregate of permeable concrete to improve the water absorption and storage performance of the permeable concrete. This research examined the influence of mineral admixture ratios on mechanical strength, capillary absorption and storage capacity, and analyzed the formation mechanisms of microporous structure. Sponge structure cement stone was prepared with a cementitious material ratio of 70% grade II fly ash, 10% slag and 20% cement. The findings indicate an optimal mix proportion that provides enhanced compressive strength, capillary water absorption, and volumetric water storage capacity. Compared with standard curing, water-bath curing was found to be unfavorable for enhancing the water absorption performance of sponge-structured cement paste; therefore, standard curing is recommended for its preparation. The pore structure of sponge-structured cement paste was analyzed using the Bruker–Emmett–Taylor (BET) method, scanning electron microscopy (SEM), Image-Pro Plus (IPP) image processing technology, and mercury intrusion porosimetry (MIP). Results indicated that the volume fraction of capillary pores in the 100–1000 nm range was positively correlated with water absorption and storage performance. The exponential relationship model between the content of grade II fly ash and the capillary pore content of sponge-structured cement stone was determined.

## 1. Introduction

In the process of sponge city construction, allowing all surface water to infiltrate into the ground may increase the burden on the city’s subsurface water storage system. To address this issue, a sponge-like cementitious material with both water absorption and retention properties—referred to as sponge structure cement paste—was developed for bonding the aggregates of pervious concrete. This approach enables the pervious concrete to allow water to pass through the large pores between coarse aggregates while its bonding material also has water absorption and storage properties, thereby overcoming the limited water retention of the existing pervious concrete [1,2,3].

In 1948, fly ash concrete was successfully applied in the Oahe Dam project in the United States, where it was found that fly ash could effectively reduce cement consumption and mitigate crack formation [4,5]. Liu et al. [6] investigated the effect of fly ash on reducing the capillary water absorption performance of concrete. Additionally, it was found that the fineness of fly ash has a significant impact on the water absorption of early-stage concrete (7 days), but in the later stage of hydration, the water absorption of concrete with different fly ash fineness gradually becomes similar, and there is no significant difference in water absorption value. Li et al. [7] reported that concrete mixed with 20% fly ash exhibited a pore size distribution that tended to be gel–gel pores, and it possesses a more homogeneous and compact microstructure. Yu et al. [8] found that under short-term curing conditions of 90 days and long-term curing conditions of 2 years, the porosity of cement slurry containing fly ash was greater than that of pure cement slurry. Young et al. [9] studied the pore size distribution of cement paste at different ages with varying slag contents and found that, at early ages, the porosity increased as the slag content was elevated. The study by Gorce [10] demonstrated that the higher the blast furnace slag incorporated, the higher the water content in the capillary pores. These findings indicate that the contents of fly ash and slag have a significant influence on the formation of pore structures and the water absorption properties of cement paste [4,8,9,11,12,13,14]. However, existing studies have primarily focused on reducing the formation of pores, while research on the mechanisms of pore formation remains scarce.

Domestic and foreign scholars have conducted extensive research on the capillary water absorption performance of cement-based materials. Hall et al. conducted early research on the water absorption characteristics of porous media under capillary adsorption [15]. Their experiments demonstrated that the capillary water uptake per unit area exhibits a strong linear relationship with the square root of the absorption time. Hall described this one-dimensional water intrusion as a linear equation of the square root of water absorption time, namely the “time square root law,” which provides a theoretical basis for future research on capillary water transport in cement-based materials. As the research progressed, it was found that in the initial stage of water absorption, the capillary water absorption per unit area of cement-based materials showed a linear growth trend with the square root of time. However, once the capillary transport reached a certain extent, the capillary adsorption gradually diminished, leading to a reduced water absorption rate and a deviation from the initial linear behavior [16]. Martys et al. [17] found in their study on the capillary transport of water in mortar and concrete that in the early stage of the capillary water absorption test, the two cement-based materials conformed to the “square root of time law”. Nevertheless, over extended periods of water transport, the capillary suction effect progressively weakened, resulting in a slower absorption rate and a transition to nonlinear growth. There are many reasons for the decrease in capillary water absorption rate in the later stage, including external environmental factors such as the initial moisture content of the material, the influence of gravity, and water evaporation. From a kinetic perspective, cement-based materials undergo further hydration, with most of the water being transported through gel pores. However, the transport speed differs from that of capillaries by an order of magnitude of 10^3^. There are many reasons for the decrease in capillary water absorption rate in the later stage, including external environmental factors such as the initial moisture content of the material, the influence of gravity, and water evaporation. From a kinetic perspective, cement-based materials undergo further hydration, with most of the water being transported through gel pores. However, the transport speed differs from that of capillaries by an order of magnitude of 10^3^. Meanwhile, some water is also consumed during the hydration process. Hall [18] first modified the original equation by adding a constant term and a linear expression for time. Wittman et al. [19] proposed a capillary water absorption model for materials with a long water absorption time. Although the accuracy has been improved to a certain extent, it was found through fitting calculation that there is still a slight gap with the original data points. Shen et al. [20] fully considered the possibility of water diffusion in the later stage of capillary water absorption, combined the gas phase diffusion phase on the basis of the pre-absorption model, and finally obtained a more accurate expression through the Fourier transform or Laplace transform. Zhang et al. [21] used neutron imaging technology to visualize the capillary water absorption process of concrete, and used three existing models to fit the measured values to quantify the spatial distribution law of water intrusion. The experiment found that the curve fitting exhibited strong agreement with the measured data within the first 72 h; however, the fitting accuracy declined thereafter. The experiment also concluded that there was a linear relationship between the capillary water absorption coefficient and the capillary coefficient of the sample, and proposed a feasible decay model of the permeability coefficient of cement-based materials according to the hyperbolic variation. Existing research mainly focuses on capillary water absorption performance, while the investigations into the water storage performance and the underlying mechanisms governing pore structure formation remain limited.

This study focuses on the use of fly ash and slag particle size as main parameters to regulate the pore structure of cementitious materials, with the aim of designing a sponge structure cement paste with superior water absorption and storage properties. By integrating microstructural pore analysis, the microscopic mechanisms underlying the macroscopic performance of a sponge structure cement paste are elucidated. The findings of this research can be applied to bonding projects for pervious concrete and high-adsorption concrete aggregates with higher water absorption and storage requirements, such as slope protection, vegetative cover, and open-graded asphalt pavements.

## 2. Raw Materials and Test Methods

### 2.1. Raw Materials

**Figure 1:**
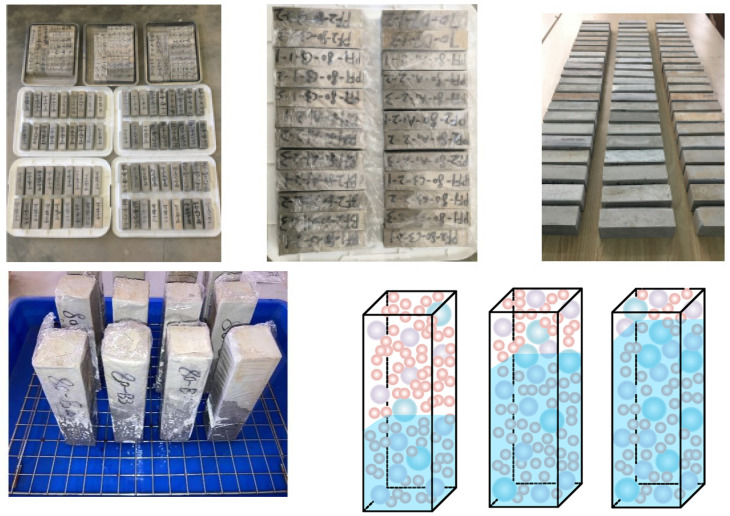
Water Absorption and Storage Test.

The raw materials for the test are as follows: P·II42.5 Portland cement produced by Qinhuangdao Qianye Cement Plant; Grade II fly ash produced by Suizhong Thermal Power Plant in Qinhuangdao; S95 slag, as shown in Figure 1; and S105 slag. The median diameters of each material were measured using the BT-9300H (Bettersize, Dandong, Liaoning, China) laser particle size analyzer, as presented in Table 1.

### 2.2. Mix Design and Specimen Preparation

This experiment adopts the method of replacing cement with an equal amount of mineral admixtures, considering the cumulative particle size distribution curves of fly ash and slag for particle grading optimization. Taking strength and fluidity as the primary design considerations, and building upon previous research [22,23], the proportion of mineral admixtures was adjusted in a gradient of 20%, accounting for 80% of the total. The water-to-binder ratios were designed as 0.4 and 0.5, respectively, with the use of RH-1 polycarboxylate high-efficiency water-reducing agent. The specific mix design is shown in Table 2.

The specimen dimensions for the mix design were 40 mm × 40 mm × 160 mm. After molding for 24 h, the molds were removed, and standard curing was carried out. The specimens marked in parentheses in Table 2 were those cured in a water bath, which were placed into a stagnant water tank at 20 ± 2 °C after demolding, with the water changed every 24 h. Twelve test blocks were formed for each mix proportion. Three specimens were selected as a group for each test. The capillary water absorption performance test data curve was selected from the curve with the saturated water mass of the three samples as the median value. The curing period was 60 days (due to the pozzolanic reaction of the ultra-high amount of mineral admixtures, the strength and pore structure of the cement paste tended to stabilize at 60 days).

### 2.3. Experimental Methods

The compressive strength of the cement paste was tested in accordance with GB/T 17671-1999 [24]. The capillary water absorption test was conducted following the ASTM C 1585-04 standard [15]. The molded specimens were dried in a constant-temperature oven until a constant weight was achieved. Four sides of the test blocks were sealed with plastic wrap to ensure that capillary water absorption occurred in a one-dimensional direction. The water absorption per unit area of the sample was denoted as *i*.

The porosity of the sponge structure cement paste was measured based on the water saturation method. Assuming the porosity of the cement paste when saturated is *P*, the relationship is as follows [25]:

(1) $$P=\frac{V_{p}}{V}=\frac{m_{1}-m_{3}}{m_{1}-m_{2}}$$

In the formula, *m*_1_ represents the saturated surface-dry mass of the test block in air; *m*_2_ represents the mass of the saturated test block immersed in water; *m*_3_ represents the mass of the test block after drying; *V_p_* represents the total volume of the open pores in the specimen; and *V* represents the total volume of the test block.

The test blocks, with a curing age of 60 days, were cut into pieces with a thickness of 1 mm and a length and width of 4 mm. The microstructure morphology of the cross-section of the samples was observed using the Hitachi S-3400N SEM (Hitachi, Tokyo, Japan) [26]. The porosity of the test blocks was analyzed using ImagePro Plus software 6.0. The micro-pore structure was investigated by nitrogen adsorption using a Micromeritics ASAP 2460 Version 3.00 instrument (Micromeritics, Norcross, GA, USA), and the pore size and pore volume curves were calculated using the BJH model. The test blocks were cut into samples of 3–6 mm and immersed in an anhydrous ethanol solution for 48 h to terminate hydration. The ethanol solution was replaced every 24 h [27]. The Quantachrome Mercury Intrusion Poremaster33 mercury porosimeter was used for mercury intrusion testing. The maximum intrusion pressure was 205 MPa, the contact angle was 140°, the mercury surface tension was 483 × 10^−3^ N/m, and the pore size ranged from a maximum of 256.26 μm to a minimum of 7.19 nm.

Mindess and Young suggested that pores smaller than 10 nm do not contribute to the transport of water and ions [4,28]. Therefore, in this study, the pore structure of the sponge structure cement paste is investigated starting from a pore size of 10 nm, with the main design focus on high water-absorbing pores with diameters greater than 100 nm [14].

## 3. Experimental Results and Analysis

### 3.1. Absorption and Retention Test

#### 3.1.1. Compressive Strength

**Figure 2:**
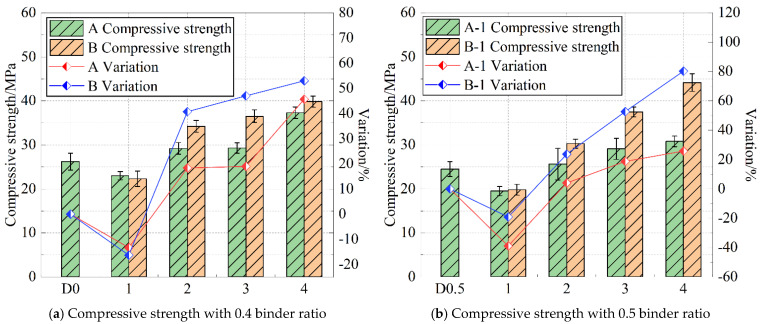
Specimen compressive strength.

Numerous studies have indicated [29,30,31,32] that the compressive strength of cement paste is closely related to its micro-pore structure, which is further regulated by the particle size combination of the material. Figure 2 presents the influence of different mineral admixtures on the compressive strength of sponge structure cement paste.

With the decrease in the dosage of Class II fly ash and the increase in the dosage of slag, the compressive strength values of specimens in Groups A and B exhibited an increasing trend. At this stage, the hydration and filling effects of slag gradually became evident, leading to a denser cement paste structure. Compared with Group A, the compressive strength values of specimens with a ratio of 1 to 4 in Group B increased more significantly. The possible reason is that the median diameter of S105 slag is 9.057 μm, which is only about half of the median diameter of Grade II fly ash, allowing it to more effectively fill the internal pores of the material and thereby improve its compressive strength.

#### 3.1.2. Capillary Water Absorption Performance

**Figure 3:**
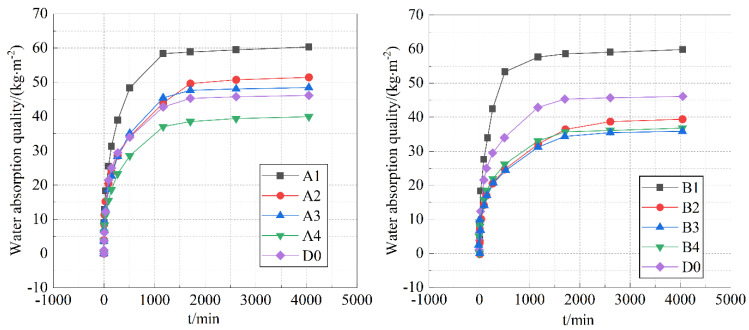
Capillary water absorption curve of 0.4 water binder ratio specimens.

Under the condition of ensuring a compressive strength of over 20 MPa, the following discussion mainly focuses on specimens with a water-to-binder ratio of 0.4. In Figure 3, specimens A1 and B1 exhibit the highest unit area water absorption mass. As the dosage of Grade II fly ash decreases, the water absorption mass shows a decreasing trend.

The water absorption phase from 0 to 270 min is the rapid absorption stage of the cement paste. During this phase, the capillary water absorption mass per unit area (i) of the cement paste exhibits a nearly linear relationship with t1/2, which follows the square root of time formula [33]. The water absorption phase from 270 to 1710 min is the transitional absorption stage. During this phase, the slope of the water absorption curve gradually decreases, and the absorption rate gradually reduces. The water absorption phase from 1710 to 4050 min is the stable absorption stage. As shown in Figure 3, increasing the content of Grade II fly ash improves the material’s water absorption rate. This is likely because high-content Grade II fly ash forms a discontinuous gradation within the material, with a larger median diameter and lower activity, resulting in larger pores that facilitate moisture absorption. Compared to S95 slag, S105 slag has a larger specific surface area, higher activity, and its hydration products significantly fill the pores within the structure, thus reducing the material’s water absorption capacity. In this phase, the absorption curve tends to level off, the water absorption mass per unit area gradually approaches zero, and the absorption reaches equilibrium.

#### 3.1.3. Influence of Curing Method

**Figure 4:**
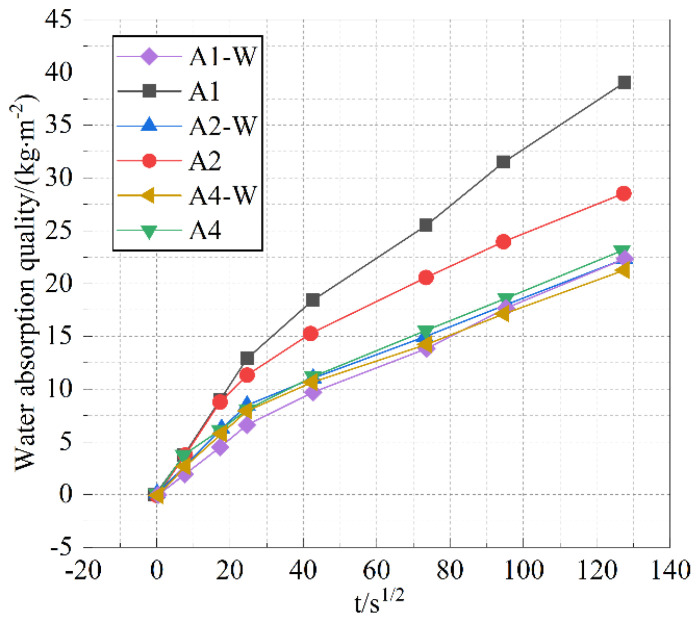
Water absorption quality curve during rapid water absorption under different curing methods.

After the addition of water to the cementitious materials, different hydration conditions can affect the hydration rate and degree of hydration of the cement paste, which in turn has a significant impact on its microstructure and macro-performance [34,35]. The effect of water bath curing and standard curing methods on the capillary water absorption performance of specimen A is shown in Figure 4.

Based on the above data analysis, it can be seen that when the content of Class II fly ash is 50% and the content of slag is 30%, the different curing methods have the most significant impact on the water absorption rate of cement stone.

As shown in Figure 3, compared to standard curing, water bath curing significantly reduced the capillary water absorption coefficients of samples A1 and A2, while having little effect on the other samples. The reason for this is that due to significant differences in the median diameter and dosage among the components, different pore structures will ultimately form in the mixture. The initial structure of cement paste is influenced by factors such as the number of particle size components and the interparticle size ratio. Appropriate physical models are selected for pore structure calculation based on different particle size combinations. Common physical packing models for multi-size regular spherical powders include the Horsfield model and the Hudson model. The final workability of cement paste is closely related to the initial structure and hydration rate of the cement paste. Upon addition of water to the mixture, it infiltrates the internal pore spaces. Part of the water is used for the hydration of the cementitious materials, and the other part ensures the good workability of the cement paste. During water bath curing, the sample rapidly absorbs water under its own capillary adsorption and hydrostatic pressure, maintaining the moisture required for cement hydration, which is more conducive to cement hydration and leads to the formation of more hydration products. During cement hydration, the volume gradually expands, filling the larger capillary pores and enhancing its density.

#### 3.1.4. Water Storage Capacity

**Figure 5:**
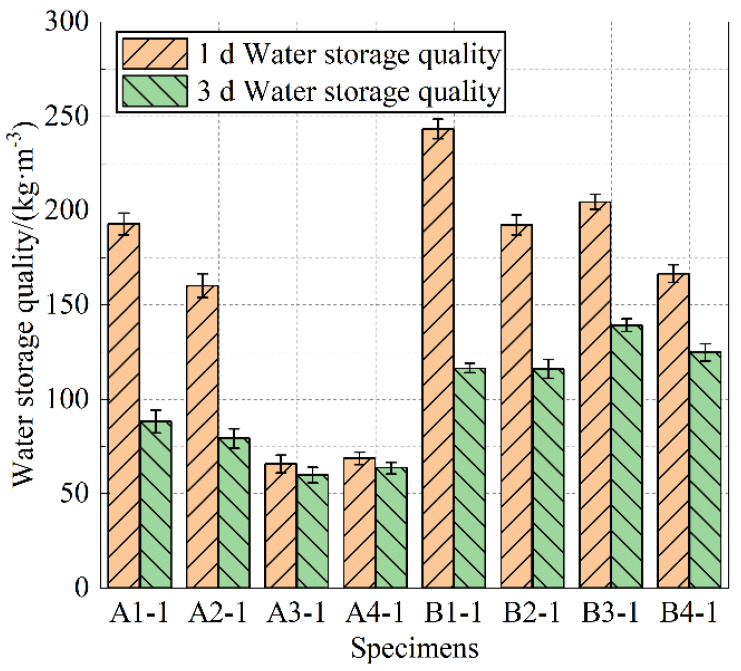
Water storage quality 1 d after water loss.

When the cement paste approaches water absorption saturation (absorption time of 4050 min), specimens from Groups A and B were placed in an indoor environment with a temperature of (23 ± 2) °C and a relative humidity of 60%. The water storage of the two groups of cement paste specimens was tested after continuous water loss for 3 days. After 24 h, the water loss rate of the specimens gradually decreased. Figure 4 presents the bar chart of the retained water mass of saturated specimens after 1 day of water loss, while Figure 5 shows the bar charts of the retained water mass of saturated specimens from Groups A-1 and B-1 after 1 and 3 days of water loss.

**Figure 6:**
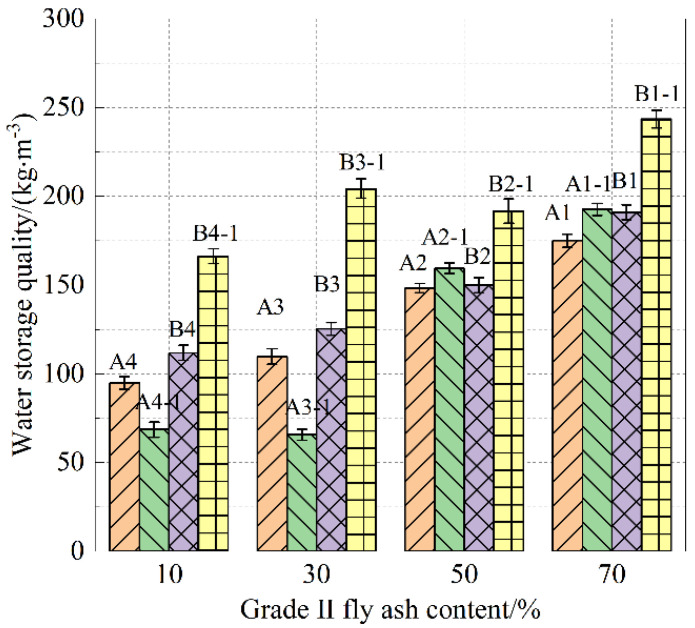
Water storage quality after water loss 1 d and 3 d.

As shown in Figure 5, with the increase in the dosage of Grade II fly ash, the water storage mass of specimens from Groups A and B generally shows an increasing trend after 1 day of water loss. Although the water-to-binder ratios differ, specimens A1 and B1 have the highest water storage within their respective groups. From the water storage comparison charts of specimens A and B after 1 and 3 days of water loss in Figure 6, it can be observed that, after 1 day of water loss, specimens A1-1 and B1-1 have the highest water storage mass, while this trend does not hold after 3 days of water loss. Given the impact of tidal and rainfall patterns on pervious concrete, the water storage capacity of the cement paste after 1 day of water loss is emphasized.

Therefore, it is concluded that when the dosage of Grade II fly ash is 70%, the water absorption and water storage performance of cement paste specimens A1 and B1 are relatively optimal. If applied to the bonding of coarse aggregates of pervious concrete, it would be more beneficial for the water purification and vegetation growth in pervious concrete, thus achieving the design goal of sponge-structured cement paste. From an economic perspective, A1 offers better prospects for engineering applications due to its cost-effectiveness.

### 3.2. Microscopic Pore Structure Analysis

#### 3.2.1. Analysis of Pore Shape

**Figure 7:**
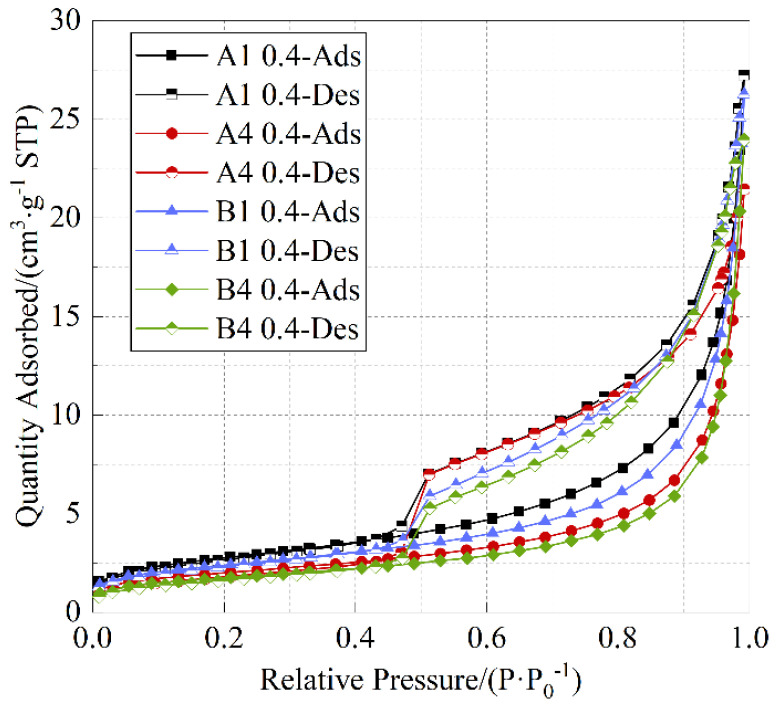
Adsorption–desorption isotherm.

The pore structure and distribution characteristics of cement paste were analyzed by nitrogen adsorption tests. Figure 7 presents the adsorption–desorption isotherms of cement paste. As shown in Figure 7, the adsorption–desorption isotherms exhibit distinct hysteresis loops. According to the hysteresis loop types recommended by the International Union of Pure and Applied Chemistry (IUPAC) [36], the hysteresis loops of the specimens are all of the H3 type, indicating the presence of parallel plate-like fracture pores within the specimens. The adsorption–desorption isotherms nearly coincide at lower relative pressures, suggesting that most of the small pores in the specimens are closed-end, non-permeable pores. At a medium relative pressure of around 0.5, a pronounced inflection point is observed, which indicates the existence of tubular capillary pores with both ends open. At higher relative pressures near 0.9, the adsorption–desorption isotherms of specimens A1 and B1 vary more significantly than the others, indicating a reduction in large pores in the remaining specimens. As shown in Figure 7, under any given relative pressure, the adsorption–desorption curve of specimen A1 remains higher than that of the others. This phenomenon demonstrates that specimen A1 contains more large pores and a relatively higher proportion of capillary pores.

#### 3.2.2. Porosity Analysis

**Figure 8:**
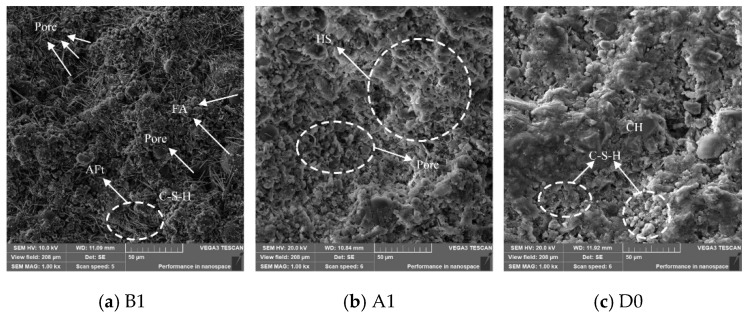
SEM image at 1000 magnification.

Microscopic analyses, including SEM and MIP, were conducted on the A1 and B1 specimens of sponge structure cement paste to further determine the characteristics of their pore structure and analyze the impact of their microstructure on macroscopic performance. Figure 8a–c present the 1000× magnification SEM images of the B1, A1, and pure cement D0 specimens at 28 days of curing.

In the figures, Pore denotes pores, FA represents unhydrated fly ash, AFt refers to needle-shaped ettringite crystals, C–S–H denotes calcium silicate hydrate, CH refers to calcium hydroxide, and HS indicates a honeycomb-like structure. As shown in Figure 8a, after 28 days of hydration, the B1 specimen contains a relatively large amount of needle-shaped AFt products. Some large particles are not tightly bonded with the surrounding substances, resulting in a loose structure with wide gaps between hydration products and the presence of larger pores. In Figure 8b, the A1 specimen exhibits a loose and porous structure with a distinct “honeycomb-like” morphology, characterized by numerous capillary pores. Most of the C–S–H appears in clustered form, with a small fraction of fibrous hydration products interspersed within. From Figure 8c, it can be seen that the cement paste structure is significantly denser. Flocculent hydration products of C–S–H gel are formed on part of the surface of Ca(OH)_2_ and embedded into the gaps. The intergrowth of layered and flocculent products enhances the compactness of the overall structure.

**Figure 9:**
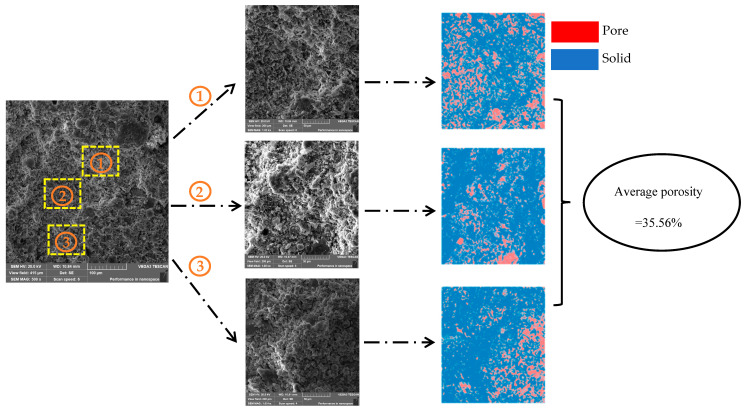
Calculating porosity.

For specimen A1, three specimens were observed at different magnifications to examine pore characteristics. To reduce errors, three test regions were randomly selected on the surface of each specimen during the actual testing, and each of these regions was analyzed separately. Under the 1000× magnification images, the unhydrated particles, hydration products, and pore areas can be clearly distinguished, providing a good representation of the pore characteristics of the sample [37]. The porosity of the specimen was calculated using Image-Pro Plus. The threshold was repeatedly adjusted until the pore structure within the solid could be just distinguished, after which noise reduction processing was applied to the image. Since the acquired images inevitably exhibited issues such as indistinct contrast and polarization, each SEM image was first converted into an 8-bit grayscale image, followed by image processing in terms of contrast, brightness, and gamma adjustments to differentiate between solid particles and pores. Subsequently, color calibration was performed through three channels with the following parameters: hue: 0–30, saturation: 0–255, and intensity: 0–255. The calculated porosity was 35.56%, and the detailed calculation process is shown in Figure 9. The same method was then used to calculate the porosity of regions (2) and (3) under 1000× magnification. Thereafter, the other two samples of specimen A1 were subjected to porosity calculations in the same manner, and the average of the nine obtained values was taken as the final porosity of specimen A1, yielding a final porosity of 34.62%.

**Figure 10:**
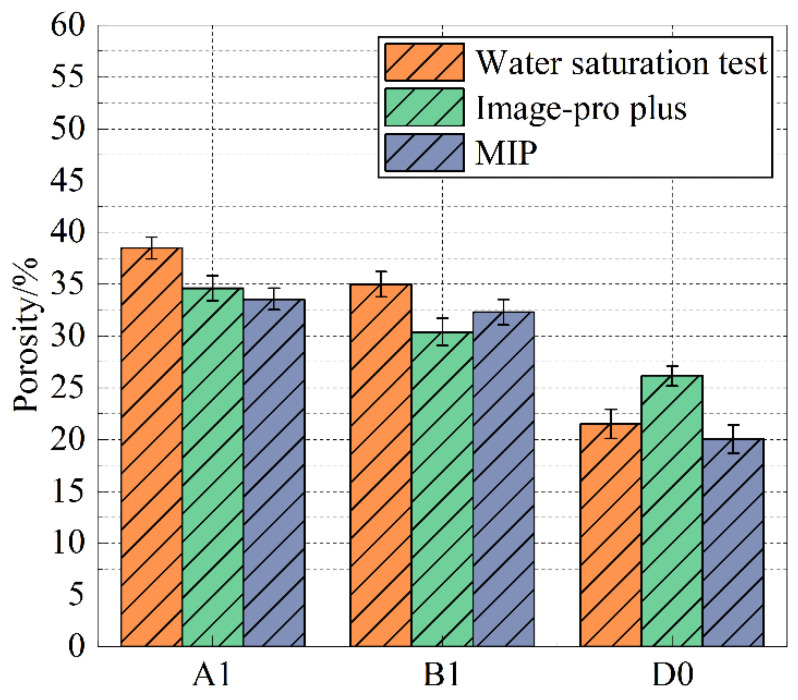
Comparison of three methods for calculating porosity.

The porosity of specimens B1 and D0 was determined sequentially. Simultaneously, the results of the water saturation method and MIP porosity tests were combined to plot Figure 10. Compared to D0, the average porosity of A1 (the average value of the three porosity test results) increased by 57.50%, while B1 showed an increase of 44.22%.

#### 3.2.3. Pore Size Distribution

**Figure 11:**
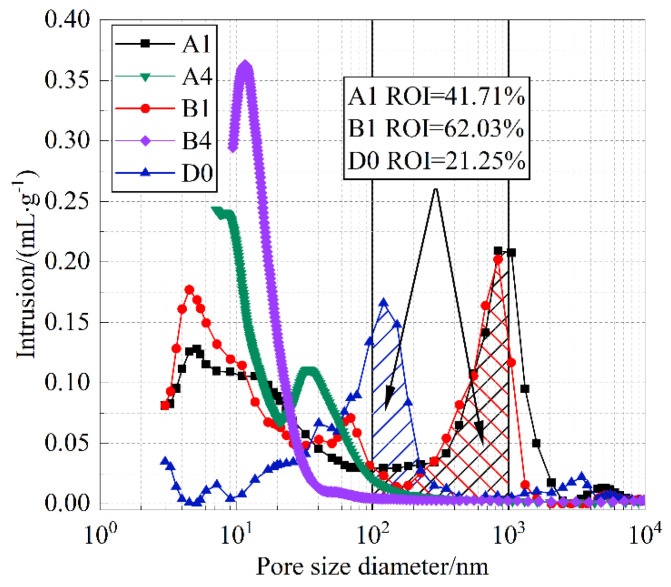
Pore size distribution curves.

According to different classification criteria of pore structures, the proportion of each pore level in the total pore volume can be determined, thereby providing a better microscopic mechanism for explaining the differences in the capillary water absorption performance of the materials. Figure 11 shows the mercury intrusion porosimetry curves of A1, B1, and D0 specimens, where the pore size distributions of A1 and B1 exhibit similarity. Based on the Barrett–Joyner–Halenda pore structure model [38], the pore size range of capillary pores is 100–1000 nm. Assuming that the ROI value represents the ratio of the pore volume within this range to the total pore volume of the specimen, the ROI values of A1, B1, and D0 are 41.7%, 62.03%, and 21.25%, respectively. It can thus be seen that the favorable water absorption capacity of A1 and B1 specimens is closely related to their higher capillary pore content. The transport of water inside cement paste includes both gas-phase and liquid-phase migration. Larger capillary pores facilitate liquid-phase water conduction. When the internal water content of the material approaches the porosity value, the contribution of gas-phase diffusion becomes non-negligible [38], and the smaller the pore size, the greater the contribution. Therefore, a broader pore size distribution is more favorable for water transport within the material. The D0 specimen exhibits a relatively narrow pore size distribution, with pores mainly concentrated around 100 nm, and containing fewer large and small pores. This indicates that the hydration degree of D0 is higher than that of B1 and A1, leading to more hydration products and lower porosity. Compared with A1 and B1 specimens, most pores in A4 and B4 specimens fall within the range of transitional pores, with pore sizes primarily between 10 nm and 100 nm, resulting in weaker capillary absorption performance. This is because a large amount of slag was incorporated into A4 and B4 specimens, which reduced the average particle size of the mixture and refined the initial porosity of the cement, thereby causing the pore sizes in the cement paste to become smaller, and shifting the pore size distribution curves and their peaks to the left.

### 3.3. Analysis of Pore Formation Patterns in Sponge Structure Cement Paste

**Figure 12:**
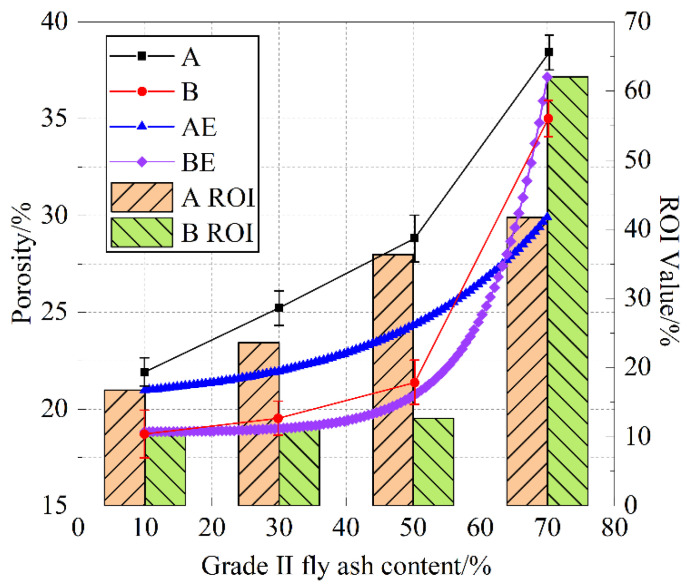
Variation trend of specimen porosity with grade II fly ash content.

From Figure 12, it can be seen that the average porosity of the A and B groups of cement paste shows a positive correlation with the addition of Grade II fly ash. Below a 50% dosage, the average porosity is almost linearly related to the fly ash content, while a 70% fly ash dosage causes a rapid increase in the average porosity. Compared to group A specimens, group B specimens show relatively lower porosity, indicating that the particle size of S95 slag, relative to S105 slag, is more suitable for blending with Grade II fly ash, promoting pore formation in sponge structure cement paste. The ROI values show that the average porosity of cement paste with different fly ash contents tends to approach similar growth rates in ROI. The ROI values can be predicted based on the macroscopic porosity test results to characterize the capillary pore (100–1000 nm) water absorption performance of cement paste. Grade II fly ash, with a larger average particle size and smaller specific surface area, belongs to a discontinuous gradation of cementitious materials, and its high dosage is the main reason for the formation of water-absorbing and water-storaging (capillary) pore sizes.

From AE and BE in Figure 12, the addition of Grade II fly ash in the A and B mixes shows an exponential function relationship with the capillary pore proportion (ROI).

(2) ROI=m+aexp[(x−b)/t]

In the equation, the values of the coefficients for group A are *m* = 14.683, *a* = 2.027, *b* = 10, and *t* = 23.164; the values of the coefficients for group B are *m* = 10.654, *a* = 0.056, *b* = 10, and *t* = 8.785.

The micro-aggregate effect of slag intensifies with the increase in its dosage. As the slag content increases, the particle spacing decreases, and the superimposed effect becomes stronger. According to the central particle theory [8], this leads to the slag filling and refining the pores in the cement paste, forming a stable pore structure that facilitates the generation of strength in sponge structure cement paste. In summary, the average pore size and capillary pore size of the sponge structure cement paste can be designed, achieving a broad-spectrum adjustable pore structure in cement paste.

## 4. Conclusions

(1) The sponge structure cement paste, prepared with a cementitious material mix of 70% Class II fly ash, 10% slag, and 20% cement under standard curing conditions for 60 days, exhibits optimal water absorption and storage properties. The compressive strength of the prepared cement paste reaches 23.7 MPa, with a capillary water absorption of 60 kg/m^2^ per unit area. After 1 day of water loss at a temperature of 23 ± 2 °C and 60% relative humidity, the water storage is 175.1 kg/m^3^, and the average porosity reaches 37.6%.

(2) Compared to standard curing, water bath curing has an adverse effect on the water absorption coefficient of cement stone in the rapid water absorption stage of sponge structure, which is not conducive to improving the water absorption performance of sponge structure cement stone. Standard curing should be used to prepare the sponge structure cement stone. During the water absorption process of cement stone in a sponge structure, in the water absorption time stage of 0–270 min, the capillary water absorption mass per unit area i of the cement stone is basically linearly related to t^1/2^. The interval from 270 to 1710 min represents the transition phase of water absorption, characterized by a gradual decline in the absorption rate. Subsequently, from 1710 to 4050 min, the water absorption process enters a stabilization stage, during which the absorption curve tends to plateau, with the water absorption mass per unit area asymptotically approaching zero and the system attaining an equilibrium state. The non-capillary pore type of sponge-structured cement stone is parallel plate-shaped fissure pores, while tubular capillary pores with open ends also exist. The ROI value of sponge-structured cement stone can reach up to 62.03%, and the relationship between fly ash content, porosity and ROI was obtained. According to the Butter pore structure model, the capillary pore size ranges from 100 to 1000 nm. The good water absorption of sponge-structured cement stone is closely related to its high capillary content. Based on this, the pore structure of sponge-structured cement stone can be synergistically controlled according to the results of fly ash and slag particle size analysis and porosity tests.

## Figures and Tables

**Table 1:** Chemical composition of cementitious materials and the median diameters.

Material	CaO/%	SiO_2_/%	Al_2_O_3_/%	SO_3_/%	Fe_2_O_3_/%	K_2_O/%	Density/ kg·m^−3^	Specific Surface Area/m^2^·kg^−1^	Median Diameter/ μm
Cement	63.40	20.20	4.95	3.33	3.32	0.94	2994	474.0	14.160
Grade II fly ash	4.48	42.70	44.80	0.61	2.31	1.14	2144	420.4	18.050
S95 Slag	32.30	32.90	17.40	2.81	0.69	0.61	2854	461.1	14.480
S105 Slag	37.70	30.60	17.40	2.44	0.41	0.51	3002	614.8	9.057

**Table 2:** Standard curing cement paste mix ratio.

Specimens	Cement/%	Grade II Fly Ash/%	S95 Slag/%	S105 Slag/%	W/B	Water-Reducing Ddmixture/%
A1(A1-W)	20	70	10	—	0.4	0.2
A2(A2-W)	20	50	30	—	0.4	0.2
A3(A3-W)	20	30	50	—	0.4	0.2
A4(A4-W)	20	10	70	—	0.4	0.2
B1(B1-W)	20	70	—	10	0.4	0.2
B2(B2-W)	20	50	—	30	0.4	0.2
B3(B3-W)	20	30	—	50	0.4	0.2
B4(B4-W)	20	10	—	70	0.4	0.2
D0(D0-W)	100	—	—	—	0.4	0.2
A1-1(A1-W-1)	20	70	10	—	0.5	0.2
A2-1	20	50	30	—	0.5	0.2
A3-1	20	30	50	—	0.5	0.2
A4-1	20	10	70	—	0.5	0.2
B1-1	20	70	—	10	0.5	0.2
B2-1	20	50	—	30	0.5	0.2
B3-1	20	30	—	50	0.5	0.2
B4-1	20	10	—	70	0.5	0.2
D0.5	100	—	—	—	0.5	0.2

## Data Availability

The original contributions presented in this study are included in the article. Further inquiries can be directed to the corresponding author.
